# Agricultural Farm-Related Injuries in Bangladesh and Convenient Design of Working Hand Tools

**DOI:** 10.1155/2018/4273616

**Published:** 2018-02-25

**Authors:** M. S. Parvez, M. M. Shahriar

**Affiliations:** Department of Industrial Engineering and Management, Khulna University of Engineering & Technology, Khulna 9203, Bangladesh

## Abstract

Injuries during cultivation of land are the significant causes of recession for an agricultural country like Bangladesh. Thousands of tools are used in agricultural farm having much probability of getting injury at their workplaces. For the injury prevention, proper hand tool designs need to be recommended with ergonomic evaluations. This paper represents the main causes of agricultural injuries among the Bangladeshi farmers. Effective interventions had been discussed in this paper to reduce the rate of injury. This study was carried out in the Panchagarh district of Bangladesh. Data on 434 agricultural injuries were collected and recorded. About 67% injuries of all incidents were due to hand tools, and the remaining 33% were due to machinery or other sources. Though most of the injuries were not serious, about 22% injuries were greater than or equal to AIS 2 (Abbreviated Injury Scale). The practical implication of this study is to design ergonomically fit agricultural hand tools for Bangladeshi farmers in order to avoid their injuries.

## 1. Introduction

A majority of workforce in the world is involved in agricultural activities [1]. In Bangladesh, around 45.1% of its population is associated with agricultural work [2]. In the field of agriculture in Bangladesh, there is also a major participation of women, not only of men. In 2010, 64.84% of the employed women in Bangladesh were found to be engaged in the agriculture sector [3]. Agricultural labor is physically demanding. The laborers engaged in agricultural works often face serious injuries at their work, like cuts on the limbs, scraping off of skin, blisters on the skin, superficial vein and deep vein cuts, cuts on toes or fingers, permanent loss of any body part, and also different musculoskeletal disorders for repetitive-type works. The injury rate in farming is the highest among all other occupations [4], and farming is also the most hazardous occupation worldwide [5]. Agronomy has consistently been identified as the major division with the highest risk of occupational skin diseases [6]. Musculoskeletal disorders are the most common of all nonfatal occupational injuries and illnesses for farmers, especially those who are involved in regular labor-intensive practices [7]. In a statistics, it was shown that in Bangladesh, a little over one quarter (26.5%) of all occupational injuries were of farmers and agricultural day laborers [8]. But all the existing sources of injury information in Bangladesh bear some deficiencies, such as lack of representativeness, low prevention orientation, and poor sustainability. The main reason of this poor surveillance system is the fact that the farmers usually are not willing to report their accidents. They are accustomed to hard work and accept the injuries as a part of their job [9]. It has been found in another study that in Bangladesh, only 35% of the injury cases were brought to the hospital for treatment [10]. The real picture of occupational injuries in Bangladesh can be inferred if we take into account the regular media reports or hospital records concerning workplace accidents. In this case, periodic household surveys on injury can be beneficial to develop the surveillance system [11]. That is why for collecting injury data, household surveys are recommended.

Workplace injuries not only incur ill health, disability, and death but also have several negative economic consequences [12]. Occupational health and safety in Bangladesh should be a higher priority in the alleviation of extreme poverty as the economy of Bangladesh is based on agriculture [13]. In a developing country like Bangladesh, the public healthcare system is usually very poor. There is no insurance and social safety because 17.6% of its population live below the poverty line [14]. So the injury victims and their families have to cover their health expenses by their own means [15]. One study found that victims in rural Bangladesh spent an average US $4 on each injury irrespective of the severity of injuries [16]. Sometimes these injuries occur repeatedly for the same person. The frequent injury causes complete physical disability of the workers. The injured laborer cannot attend to his or her work. Agricultural workers spend 42 hours per week on average at their workplaces; in most cases, they are the only earning member of their family [2]. Occupational injuries thus increase the risk of extreme poverty. Occupational accidents can be prevented by implementing the available measures and methods which will eliminate the factors that are causing the accidents [17]. Handle diameter and handle length are the key factors to minimize workplace injuries [18].

Finding optimum tool handle dimensions by using hand anthropometry has been the most common [19–22] and effective way for hand tool injury reductions. Ergonomic principles have already been used successfully to control injuries for road accidents, industry, and sports; but their application in agriculture is limited. According to Fenske and Simcox [23], ergonomic strain associated with agricultural work can be minimized or entirely prevented by redesigning the farm equipment and labor practices. Every day, some new agricultural equipment without ergonomic considerations and design is being introduced in the market. Most of the existing hand tools are made by a particular group of people who conduct tool business and manufacture them by following traditional design and materials with lack of ergonomic concepts. This study has focused on understanding the mechanism of hand tool injuries in traditional farming activities and controlling the rate of injury through ergonomic study of hand tools.

## 2. Materials and Methodology

This study was done in three different steps.

### 2.1. Survey of Agricultural Injuries

Data on agriculture-related injuries were collected from the Panchagarh district of Bangladesh. Survey areas were located through multistage cluster sampling method. There are a total of five upazilas (subdistricts), including a total of 843 villages in the Panchagarh district. Among the five upazilas, the Boda and Debiganj upazilas were randomly selected. The number of villages in Boda and Debiganj upazilas is, respectively, 239 and 100. Then, all the villages were clustered, and 235 villages were randomly selected among 339 villages for our injury survey purpose. These villages are predominantly rice, sugarcane, and jute growing areas. The villages were selected for high agricultural involvement and absence of industry. In all these areas, cultivation methods and the quality of medical facilities were the same. Six field workers were trained by the authors to collect injury data from household surveys. The field workers were selected locally for the convenience of our study. It took seven months and two weeks for the field workers to complete their survey. Victims were interviewed regarding injury- and equipment-related information. A total of 434 injuries were found and recorded.

Standard interview was conducted with the agricultural farm workers by taking full permission from the interviewee and seeking the interviewees' free time for the purpose of reducing participant error. The interview was made short, and care was taken to ensure that the participants may not feel annoyed or bored while some open-ended structured questions were being asked. To eradicate participant bias, injured persons were interviewed when they were alone, so that their responses may not be affected by the outside environment. To make the data collection reliable, two field workers conducted the interview together. One asked questions while the other recorded the responses, thus eliminating researcher error. The field workers stopped their survey work when they felt tired and uninterested to conduct the survey further. Discussions were also made with farm workers regarding the comforts and inconveniences they deal with their hand tools. The injuries that occurred more than one time for a single person within a period of one year were also recorded. The survey was continued by the field workers until the last person who was injured in that region was visited. After the visit, the houses were marked by the field workers to eradicate the chance of a repeat household survey. The severity of injury was recorded according to the Abbreviated Injury Scale (AIS) [24]. The AIS is from 0 to 6, where 0 = no injury, 1 = minor, 2 = moderate, 3 = serious, 4 = severe, 5 = critical, and 6 = maximum (untreatable). In this study, data regarding injuries caused by agricultural implements are included. These data provided the basic guidelines for designing safer hand tools.

### 2.2. Procedure for Making Ergonomic Evaluation of Hand Tools and Recording Anthropometric Dimension of Hands of Farm Workers

**Figure 1:**
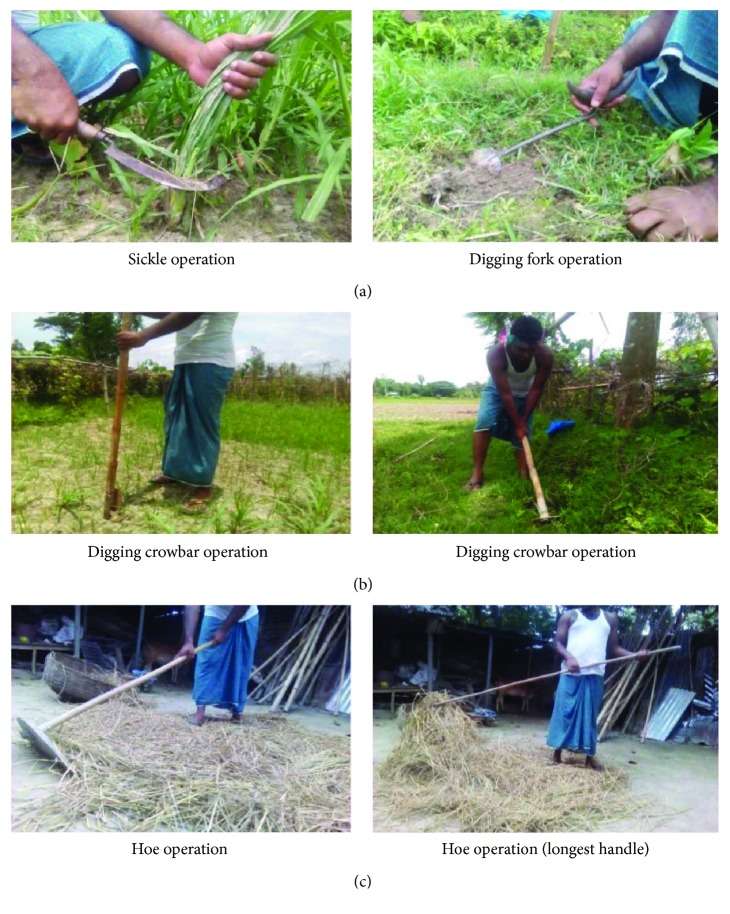
Farm hand tools. (a) Small handle, (b) medium handle, and (c) long handle.

Ergonomic evaluation of hand tools was made by measuring the dimensions of handles and taking anthropometric measurements of workers' hands. There are many types of hand tools used in Bangladeshi farms. In this study, the agricultural hand tools are divided into three categories as per their handle length shown in Figure 1.

The tools having 6–10 cm length are considered as small handle. Similarly, 75–90 cm length is for medium handle and 115–150 cm length for long handle. Small handle tools include sickles, daggers, digging forks, and small rakes; medium handle tools include axes and spades; and long handle tools include hoes and digging crowbars. Dimensions of some existing tools (handle diameter and handle length) were measured and recorded from different villages of that region.

**Figure 2:**
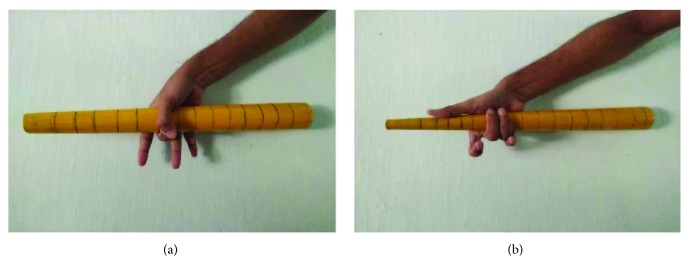
Hand anthropometric dimension measuring tools: (a) wooden conical bar to measure inside grip diameter and (b) wooden conical bar to measure middle finger palm diameter.

A total of 42 hand tools that were available in the agricultural farms were observed. Anthropometric dimensions of hands were recorded from 60 farm workers from rural areas to estimate the handle design. The measurement technique of inside grip, palm diameter, and palm width and length is shown in Figure 2. Two conical wooden bars (as used by Kumar et al. [21]) were used for measuring inside grip diameter and middle finger palm diameter. A flat board was also used for this purpose. The bare hands were kept straight on the board. Then the measurement of palm breadth across the thumb was taken using the slide calipers on the straightened hand.

### 2.3. Interventions for Hand Tool Injury Prevention

To make ergonomic interventions of hand tools, dimensions of existing hand tools were compared with critical anthropometric dimensions of hands of the farmers in that region. Proper ergonomic interventions were made by figuring out the information associated with hand tool injuries, existing hand tool design, and hand anthropometry of agricultural workers.

Interventions were made by suggesting proper handle dimensions based on anthropometric consideration, which recommends that the handle diameter for three types of agricultural hand tools should lie between inside grip diameter of the 5th percentile and 95th percentile of middle finger palm dimension and that the handle length of small handle tool should accommodate the 95th percentile of palm breadth [21].

## 3. Results

### 3.1. Record of Injuries

**Figure 3:**
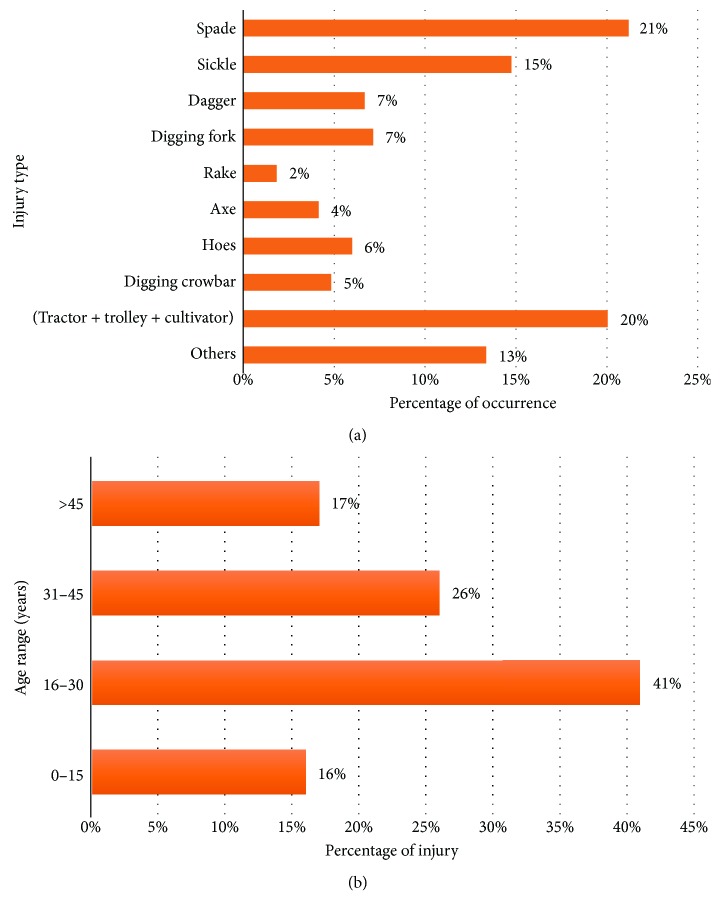
Farm-related injury (a) according to hand tools and (b) according to age.

Among all the injuries, the injuries like cuts on the skin, scraping off of skin, superficial vein cuts, cuts on toes or fingers, and muscle stresses were recorded as AIS 1 severity, whereas cuts on limbs, deep vein cuts, permanent loss of any part of the body, and infections at injured limbs were considered between AIS 2 and AIS 3 severity of injury. Among all the injuries, most were found to be minor, ranging within AIS 1 to AIS 3. AIS 4 and AIS 5 were not found, and the 2 cases of AIS 6 were too negligible. Severity by injury type of some injuries was difficult to identify. Different types of injuries are presented in Table 1, and farm-related injuries are shown in percentage in Figure 3(a). Age distribution of the victims injured by various equipment and machines is summarized in Table 2, and percentages of injuries related to different ages are depicted in Figure 3(b). These data conclude that maximum injury occurred between the ages 16 and 30 years.

### 3.2. Magnitude of Injuries

In this study, it was observed that 67% of agricultural injuries occurred due to hand tools and only 20% by cultivating machine such as trolley or tractor. Hence, hand tool injuries were predominant in this observation. Most of the injury occurred among 16- to 30-year-old workers, though 78% of the hand tool injuries were minor injuries (AIS 1), 17.5% were AIS 2, and less than 1% were AIS 6. Recovery time period was observed to be quite long as the farmers were usually reluctant to stop working during the recovery period. This tendency was due to their extreme poverty.

### 3.3. Frequency of Injury

Frequencies of injury were recorded for the persons who had experienced injuries more than one time within a period of one year, which is stated in Table 3.

### 3.4. Anthropometric Dimension of Hands and Ergonomic Evaluation of Hand Tools

Anthropometric data of inside grip diameter, middle finger palm diameter, and palm breadth of 60 people of the Panchagarh district in Bangladesh were collected and summarized in Table 4.

Based on the anthropometric consideration as discussed in Materials and Methodology, the handle diameter for three types of agricultural hand tools should lie between inside grip diameter of the 5th percentile and 95th percentile of middle finger palm dimension, and the handle length of small handle tool should accommodate the 95th percentile of palm breadth. According to this evaluation, based on hand anthropometric measurements of workers, the suggested handle diameter of hand tools should lie between 3.2 cm and 3.8 cm and handle length should not be less than 8.5 cm (Table 4).

**Figure 4:**
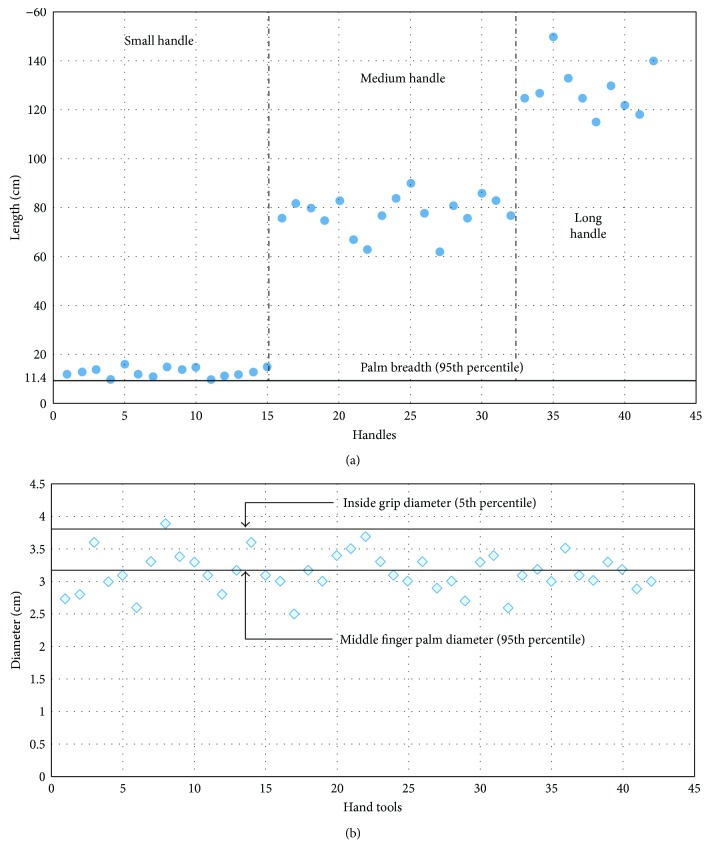
Deviation of handle dimensions with respect to suggested dimensions of (a) handle length and (b) handle diameter.

With the observation of 42 existing hand tools, the range of length and diameter of handles is found, respectively, to be 6–10 cm and 2.6–3.3 cm, 75–90 cm and 2.5–3.2 cm, and 115–150 cm and 2.6–3.4 cm. The deviations of existing tool dimensions from suggested handle diameter and handle length are shown in Figure 4, where it shows that 24 out of 42 tool dimensions were beyond our suggested tool dimension limit.

## 4. Discussion

In Bangladesh, several types of hand tools are used in the agricultural field, such as sickles, daggers, digging forks, rakes, axes, spades, hoes, and digging crowbars. The tool handles available in Bangladeshi farms are mainly made of bamboo and wood. Some causes were found out during the interview of the injury victims. A handle without a smooth surface causes blisters on the palm skin. The sickle is a hand tool which causes lots of cut injuries such as cuts on the skin, superficial vein cuts, cuts on toes or fingers, and cuts on the limbs during harvesting because of its tip's sharpness. Land preparation in agriculture is time-consuming and requires a lot of physical labor. Prolonged mode of work and continuous palm sweating cause tool slippage from hand during operation. This slippage is the leading reason for injuries like cuts on the skin, scraping off of skin, superficial vein and deep vein cuts, cuts on toes or fingers, muscle stresses, cuts on the limbs, and permanent loss of any part of body. Muscle stresses, permanent loss of any body part, and cut injuries mainly occur while working with hoes, digging forks, digging crowbars, and daggers. Sickles and hoes, which are used for removing weeds or cutting crops, injure the hand; the left hand is used for gathering and holding the crop and the right hand for holding the tool. This mode of operation causes deep cut injuries. While performing axe operation, if the target is missed, deep cuts or serious injuries can happen to the worker. This may lead to permanent loss of the injured body part.

In this study, it has been found that spades and sickles were mainly responsible for the larger proportion of injuries (21% and 15%, resp.) at agricultural farms (Table 1). Injuries caused by daggers, digging forks, digging crowbars, and hoes were less significant (within 5% to 7%) of all injuries. The amount of injuries caused by axes and rakes was found to be lesser (below 4%) than injuries caused by other hand tools (Table 1). Other injuries that occurred in farming include various animal and snake bites, heat stroke, and different side effects during or after using fertilizers or pesticides. Xiang et al. [25] found that in Hubei, China, 50% of agricultural injuries are caused by hand tools. A cross-sectional study from rural Nepal [26] indicated the hand tool as highly responsible for injuries among Nepalese farmers. Xiang et al. [25] also found that a significant number of farm injuries in India are caused by hand tools. In this study, farm machineries (tractor, trolley, and cultivator) were also found as a significant source of agricultural injury (20%) but less frequent than hand tools (Figure 3(a)). Tiwari et al. [27] reported that 77.6% of all agricultural injuries in India were due to farm machinery. No previous study has given so much interest to farm machinery as a cause of agricultural injury. Browning et al. [28] found that 22.5% of the injuries among the older Kentucky farmers were caused by machineries.

More than 40% of injuries occurred within the 16–30-year-old group people (young people), and 26% occurred within the 31–45-year-old group (Table 2). These findings are similar to those of Demers and Rosenstock [29], in which 74% of all the injury claimants were between the ages of 18 and 40. Tiwari et al. [27] found that the highest rate of injury (32.9%) was by 15–29-year-old farmers. The injury frequency was also obtained from the injury victims who have experienced injury more than one time in a year. Around 40% of the injury victims reported on experiencing repeated injuries (Table 3). However, Rautiainen et al. [30] in their survey showed that among 93,550 Finnish farmers, only 493 (0.52%) reported on experiencing their injury more than one time in a year. Therefore, it can be said that the Bangladeshi agricultural system is more unsafe and perilous than that of other countries in the world.

Accidental hazards occur due to impact type of hand tools like spades, digging forks, digging crowbars, or axes due to the unpredictable nature of soil, standing water, or blade hitting hard surfaces like those of stones. Tools with very small handle diameter can slip from hands. Handle slippage can be prevented by an appropriate shape of the handle [22]. Improper dimension of handle, improper gripping posture, improper materials, and excessive handle weight result in wrist deviations and cause musculoskeletal disorders. Sometimes the hand tools that the farmers use are homemade, which is also a considerable issue for causing agricultural farm injuries. In almost all cases, the gripping facilities are not provided with these homemade hand tools. These handles are made with available cheap materials following traditional poor design.

Hand tools bear a significant importance for ensuring workplace safety in agriculture [21]. It has also been previously shown that objects that follow the shape of hand result in much lower contact pressures of the soft tissue, which can prevent discomfort and several disorders [31]. The design of hand tools depends upon many important factors like mode of operation, anthropometric data of user population, and material and dimension of handle. Anthropometric data are a prerequisite for designing agricultural tools and equipment that enable workers to achieve better performance and productivity while providing better safety and comfort [32]. Anthropometric considerations used in designing hand tools will increase efficiency of the workers. This study has suggested handle dimensions for hand tools by following ergonomic principles (Table 4). In this study, it was observed that the existing tool dimensions were not as recommended. The comparison in Figure 4 shows that 24 out of 42 tool dimensions were not as recommended. Since the hand tools available in farms are not of proper dimensions, the rate of injury is significant in this region. Thus is can be said that the improper dimension of tool handles is the main cause of several agricultural injuries. Though all the reasons of hand tool injuries were not found out, some of these have been found and analyzed.

## 5. Conclusion

Hand tools contribute 67% of total agricultural injuries in Bangladesh. The most significant injuries were cuts on the limbs, blisters on palm skin because of high stress in hand, tool slippage from hand, and so on. The mentionable reason behind these injuries is the mismatch of anthropometric dimensions of workers' hands with measured ergonomic tool handle dimensions. Improper handle dimensions lead to high stress and injury at work and sometimes result in workers' physical disability. To achieve better productivity along with better safety and comfort, the whole working system and tools must be redesigned so that these can be suitable for the workers to use. The handle is an important part of hand tools. Thus a proper grip dimension is very important to ensure effectiveness when operating the tool. That is why anthropometric considerations are needed for such design work. This study was focused on developing the farming sector of agriculture through ergonomic principles. The result of this study may improve the design of hand tools and may inspire the manufacturers in using recommended tool handle dimensions to apply these in practice and to design hand tools or equipment that suit the physical characteristics of the workers.

## 6. Limitations and Recommendations

In this study, anthropometric data were collected from 60 male people of the Panchagarh district. Though there is not much ethnic or geographical diversity among Bangladeshi people of different regions, the authors suggest that future researchers observe a large number of sample population from different regions and design tools accordingly. As the scenario of working condition in most of the villages in Bangladesh is almost the same, the provided injury data can be a great resource for the administration and for social workers to understand the working condition in agricultural farms of Bangladesh.

## Figures and Tables

**Table 1:** Distribution of injuries by type of agricultural implements used and severity of injury.

	Implements and hand tools	Severity of injury														Total
		AIS 1				AIS 2				AIS 3				AIS 6	Unknown	
		Superficial vein cuts	Cuts on toes or fingers	Muscle stresses	Other injury type	Deep vein cuts	Permanent loss of any body part	Infection at injury limbs	Other injury type	Deep vein cuts	Permanent loss of any body part	Infection at injury limbs	Other injury type	For all injury severity	For all injury severity	
Small handle	Sickles	27	14	9	8	3	1	2	—	—	—	—	—	—	—	64
	Daggers	14	5	2	—	2	2	1	1	1	1	—	—	—	—	29
	Digging forks	9	11	4	3	2	—	1	—	1	—	—	—	—	—	31
Medium handle	Rakes	3	—	4	1	—	—	—	—	—	—	—	—	—	—	8
	Spades^∗^	26	34	6	11	9	3	2	—	—	1	—	—	—	—	92
	Axes^∗^	3	9	—	—	1	1	—	—	1	1	1	—	—	1	18
Long handle	Hoes	9	1	5	7	2	—	1	1	—	—	—	—	—	—	26
	Digging crowbar^∗^	4	11	—	1	3	1	—	—	1	—	—	—	—	—	11
Machinery and others	(Tractor + trolley + cultivator)	14	13	16	8	12	3	7	6	2	1	—	1	2		87
	Other tools	16	11	8	12	5	1	2	1	1	—	1	—	—	—	58
	Total	125	109	54	51	39	12	16	9	7	4	2	1	2	3	434

^∗^Handles can be in both long and medium range. Most of the digging crowbars have long handles, and most of the spades and axes have medium handles.

**Table 2:** Distribution of injuries by type of agricultural implements with age range of victims.

Implements	Age (years)				Total
	5–15	16–30	31–45	>45	
Hand tools (all)	45	119	71	54	289
(Tractor + trolley + cultivator)	16	37	25	9	87
Others	8	22	17	11	58
Total	69	178	113	74	434

**Table 3:** Injury frequency of farm workers.

Frequency of injury	Number of injured persons	Percentage
1	257	59.2
2	104	24
3	33	7.6
4	12	2.8
5+	28	6.4
Total	434	100

**Table 4:** Hand anthropometric dimensions (in cm) (*N* = 60).

Hand dimensions	Percentile			Minimum	Maximum	Standard deviation
	5th	50th	95th			
Inside grip diameter	3.8	4.2	4.6	3.6	4.6	0.23
Middle finger palm diameter	2.1	2.5	3.2	2.02	3.32	0.35
Palm breadth thumb	8.5	9.8	11.4	7.5	12	1.1
